# Supplementary material for: Unveiling the Bioactive Potential of the Invasive Jellyfish Phyllorhiza punctata Through Integrative Transcriptomic and Proteomic Analyses
Source: Biomolecules. 2025 Aug 4;15(8):1121. doi: 10.3390/biom15081121 (PMC12383608; doi:10.3390/biom15081121)
Supplement: Supplementary file 1 [file biomolecules-15-01121-s001.zip › Suplementary Material/FileS3/PEPFOLD4/XP_065069170_15377/clusters.html]

 PEP-FOLD clusters


Download archive of all models  here   

|  |  |  |
| --- | --- | --- |
| # | file | sOPEP |
| # cluster 1 - Size: 37 |  |  |
|  | XP\_065069170\_15377-model1 | -30.0102 |
|  | XP\_065069170\_15377-model1.1 | -29.8133 |
|  | XP\_065069170\_15377-model1.2 | -29.7741 |
|  | XP\_065069170\_15377-model1.3 | -29.752 |
|  | XP\_065069170\_15377-model1.4 | -29.5846 |
|  | XP\_065069170\_15377-model1.5 | -29.5724 |
|  | XP\_065069170\_15377-model1.6 | -29.5333 |
|  | XP\_065069170\_15377-model1.7 | -29.5272 |
|  | XP\_065069170\_15377-model1.8 | -29.5155 |
|  | XP\_065069170\_15377-model1.9 | -29.5033 |
|  | XP\_065069170\_15377-model1.10 | -29.5028 |
|  | XP\_065069170\_15377-model1.11 | -29.4988 |
|  | XP\_065069170\_15377-model1.12 | -29.4849 |
|  | XP\_065069170\_15377-model1.13 | -29.4774 |
|  | XP\_065069170\_15377-model1.14 | -29.4616 |
|  | XP\_065069170\_15377-model1.15 | -29.4466 |
|  | XP\_065069170\_15377-model1.16 | -29.4377 |
|  | XP\_065069170\_15377-model1.17 | -29.434 |
|  | XP\_065069170\_15377-model1.18 | -29.4287 |
|  | XP\_065069170\_15377-model1.19 | -29.4209 |
|  | XP\_065069170\_15377-model1.20 | -29.4161 |
|  | XP\_065069170\_15377-model1.21 | -29.4077 |
|  | XP\_065069170\_15377-model1.22 | -29.4039 |
|  | XP\_065069170\_15377-model1.23 | -29.4015 |
|  | XP\_065069170\_15377-model1.24 | -29.3853 |
|  | XP\_065069170\_15377-model1.25 | -29.3508 |
|  | XP\_065069170\_15377-model1.26 | -29.3274 |
|  | XP\_065069170\_15377-model1.27 | -29.2858 |
|  | XP\_065069170\_15377-model1.28 | -29.2485 |
|  | XP\_065069170\_15377-model1.29 | -29.2151 |
|  | XP\_065069170\_15377-model1.30 | -29.2014 |
|  | XP\_065069170\_15377-model1.31 | -29.1879 |
|  | XP\_065069170\_15377-model1.32 | -29.1473 |
|  | XP\_065069170\_15377-model1.33 | -29.132 |
|  | XP\_065069170\_15377-model1.34 | -29.033 |
|  | XP\_065069170\_15377-model1.35 | -28.6939 |
|  | XP\_065069170\_15377-model1.36 | -28.4197 |
| # cluster 2 - Size: 1 |  |  |
|  | XP\_065069170\_15377-model2 | -22.5251 |
| # cluster 3 - Size: 11 |  |  |
|  | XP\_065069170\_15377-model3 | -21.561 |
|  | XP\_065069170\_15377-model3.1 | -21.4991 |
|  | XP\_065069170\_15377-model3.2 | -21.3462 |
|  | XP\_065069170\_15377-model3.3 | -21.1438 |
|  | XP\_065069170\_15377-model3.4 | -20.8069 |
|  | XP\_065069170\_15377-model3.5 | -19.5405 |
|  | XP\_065069170\_15377-model3.6 | -18.9053 |
|  | XP\_065069170\_15377-model3.7 | -18.8235 |
|  | XP\_065069170\_15377-model3.8 | -18.8141 |
|  | XP\_065069170\_15377-model3.9 | -18.5459 |
|  | XP\_065069170\_15377-model3.10 | -18.5081 |
| # cluster 4 - Size: 11 |  |  |
|  | XP\_065069170\_15377-model4 | -21.1595 |
|  | XP\_065069170\_15377-model4.1 | -21.0411 |
|  | XP\_065069170\_15377-model4.2 | -20.9126 |
|  | XP\_065069170\_15377-model4.3 | -20.8075 |
|  | XP\_065069170\_15377-model4.4 | -20.7776 |
|  | XP\_065069170\_15377-model4.5 | -20.5195 |
|  | XP\_065069170\_15377-model4.6 | -20.4632 |
|  | XP\_065069170\_15377-model4.7 | -20.1729 |
|  | XP\_065069170\_15377-model4.8 | -20.1392 |
|  | XP\_065069170\_15377-model4.9 | -19.8236 |
|  | XP\_065069170\_15377-model4.10 | -18.4409 |
| # cluster 5 - Size: 4 |  |  |
|  | XP\_065069170\_15377-model5 | -19.8258 |
|  | XP\_065069170\_15377-model5.1 | -19.8014 |
|  | XP\_065069170\_15377-model5.2 | -19.138 |
|  | XP\_065069170\_15377-model5.3 | -18.3706 |
| # cluster 6 - Size: 16 |  |  |
|  | XP\_065069170\_15377-model6 | -18.9927 |
|  | XP\_065069170\_15377-model6.1 | -18.6819 |
|  | XP\_065069170\_15377-model6.2 | -18.6307 |
|  | XP\_065069170\_15377-model6.3 | -18.5143 |
|  | XP\_065069170\_15377-model6.4 | -18.4859 |
|  | XP\_065069170\_15377-model6.5 | -18.4238 |
|  | XP\_065069170\_15377-model6.6 | -18.2733 |
|  | XP\_065069170\_15377-model6.7 | -18.2299 |
|  | XP\_065069170\_15377-model6.8 | -18.1867 |
|  | XP\_065069170\_15377-model6.9 | -18.1396 |
|  | XP\_065069170\_15377-model6.10 | -17.9997 |
|  | XP\_065069170\_15377-model6.11 | -17.9028 |
|  | XP\_065069170\_15377-model6.12 | -17.8973 |
|  | XP\_065069170\_15377-model6.13 | -17.88 |
|  | XP\_065069170\_15377-model6.14 | -17.8072 |
|  | XP\_065069170\_15377-model6.15 | -17.6506 |
| # cluster 7 - Size: 7 |  |  |
|  | XP\_065069170\_15377-model7 | -18.6891 |
|  | XP\_065069170\_15377-model7.1 | -18.5391 |
|  | XP\_065069170\_15377-model7.2 | -18.5372 |
|  | XP\_065069170\_15377-model7.3 | -18.5288 |
|  | XP\_065069170\_15377-model7.4 | -18.5194 |
|  | XP\_065069170\_15377-model7.5 | -18.4248 |
|  | XP\_065069170\_15377-model7.6 | -17.9947 |
| # cluster 8 - Size: 2 |  |  |
|  | XP\_065069170\_15377-model8 | -18.4615 |
|  | XP\_065069170\_15377-model8.1 | -17.9456 |
| # cluster 9 - Size: 2 |  |  |
|  | XP\_065069170\_15377-model9 | -18.2786 |
|  | XP\_065069170\_15377-model9.1 | -18.1117 |
| # cluster 10 - Size: 5 |  |  |
|  | XP\_065069170\_15377-model10 | -18.2115 |
|  | XP\_065069170\_15377-model10.1 | -18.197 |
|  | XP\_065069170\_15377-model10.2 | -17.9792 |
|  | XP\_065069170\_15377-model10.3 | -17.9527 |
|  | XP\_065069170\_15377-model10.4 | -17.7952 |
| # cluster 11 - Size: 3 |  |  |
|  | XP\_065069170\_15377-model11 | -18.0573 |
|  | XP\_065069170\_15377-model11.1 | -17.7896 |
|  | XP\_065069170\_15377-model11.2 | -17.712 |
| # cluster 12 - Size: 1 |  |  |
|  | XP\_065069170\_15377-model12 | -17.9057 |
|  |  |  |
